# Bronchogenic Cyst and Pericardial Agenesis: A Case Report

**DOI:** 10.7759/cureus.67255

**Published:** 2024-08-19

**Authors:** Adriana Simoneta Pimienta-Ibarra, Pablo Gomes-da Silva de Rosenzweig, Jose L Tellez-Becerra, María J Midence-Argüello, César Luna-Rivero, Francina V Bolaños-Morales

**Affiliations:** 1 Department of Thoracic Surgery, Instituto Nacional de Enfermedades Respiratorias, Mexico City, MEX; 2 Experimental Lung Transplant Unit, Instituto Nacional de Enfermedades Respiratorias, Mexico City, MEX; 3 Department of Pathology, Instituto Nacional de Enfermedades Respiratorias, Mexico City, MEX

**Keywords:** video-assisted thoracic surgery, cardiac luxation, congenital, complex bronchogenic cyst, pericardial agenesis

## Abstract

Bronchogenic cysts are a rare congenital condition, and their association with pericardial agenesis is even more uncommon. We present the case of a 23-year-old patient who was diagnosed with an upper lobe bronchogenic cyst and subsequently underwent an upper lobectomy. Further evaluation revealed the presence of complete left pericardial agenesis.

## Introduction

Bronchogenic cysts are congenital malformations that account for 10-15% of mediastinal tumors and 50-60% of mediastinal cysts [1]. Intrapulmonary cysts make up 20-30% of bronchogenic cysts, with most located in the inferior lobes [2]. While many patients are asymptomatic, these cysts can sometimes present with recurrent pneumonia and compressive symptoms [3]. This report describes a female patient diagnosed with a symptomatic bronchogenic cyst, in whom pericardial agenesis was also discovered.

## Case presentation

**Figure 1:**
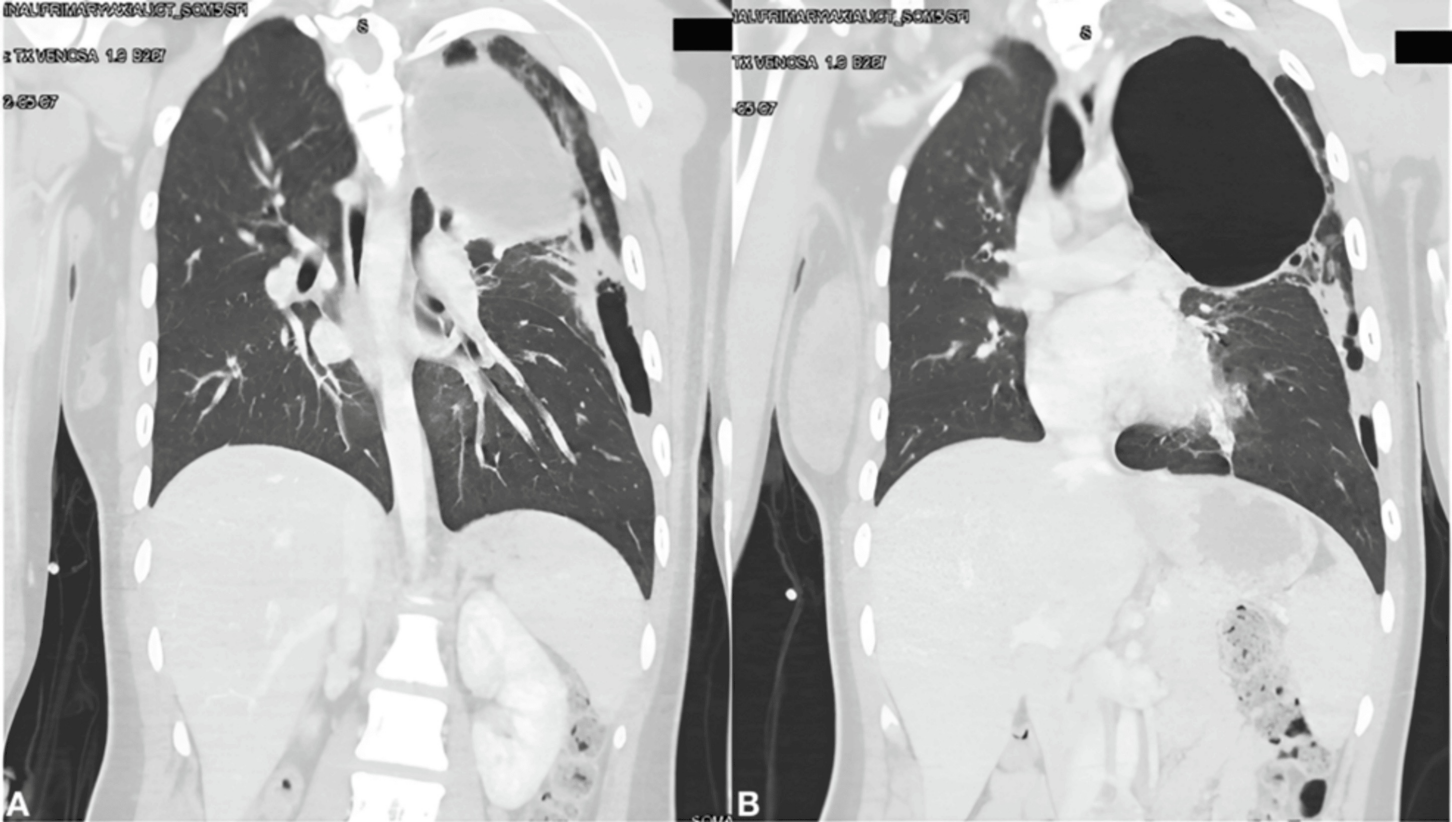
Preoperative coronal CT scan (A) Cystic lesion of 7 cm in diameter with liquid content, without communication to the airway. (B) Cavitated lesion identified laterally and inferiorly to the cystic mass.

A 23-year-old woman presented with a four-month history of progressive dyspnea and cough with purulent sputum and was initially evaluated at an external clinic. She was diagnosed with community-acquired pneumonia; however, her condition failed to improve despite adequate antibiotic treatment, leading to her referral to our institute. A CT scan revealed a cystic mass in the left upper lobe with a cavitated lesion exhibiting a hydroaerial level adjacent to it (Figure 1). These findings raised the primary suspicion of a bronchogenic cyst. Preoperative pulmonary function tests were conducted, demonstrating acceptable lung function with an FEV1 of 99.4%, an FEV1 of 2.33 L (82%), an FVC of 2.47 L (76%), and an estimated postoperative FEV1 of 1.71 L, indicating that the patient was suitable for an upper lobectomy.

**Figure 2:**
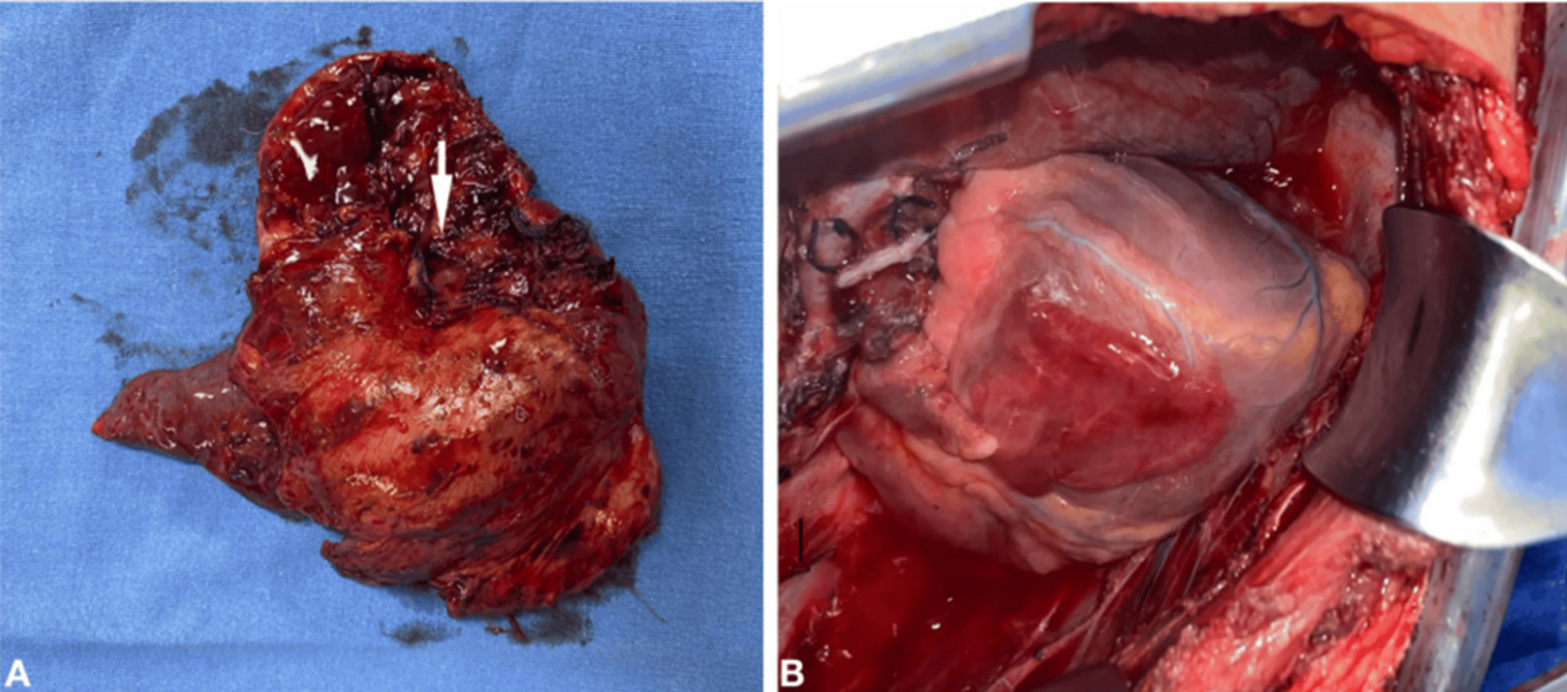
Intraoperative findings (A) Surgical specimen showing the left upper lobe adjacent to the bronchogenic cyst. (B) Left atrial appendage identified during the resection of the bronchogenic cyst.

Resection of the lesion was performed using video-assisted thoracic surgery (VATS), revealing a cystic mass measuring 7 × 3 cm that was firmly adhered to the mediastinum. The mass extended toward the thoracic wall at the apical level and involved the superior lobe (Figure 2A). During the resection, the left atrial appendage was identified (Figure 2B). Upon completion of the upper lobectomy via thoracotomy, an absent pericardial sac was observed (Figure 2B). The procedure lasted five hours, and postoperative arterial blood gas analysis showed the following results: pH 7.26, pCO2 38.6 mmHg, pO2 69.8 mmHg, HCO3 17.1 mmol/L, base excess -8.8 mmol/L, and lactate 1.6 mmol/L.

**Figure 3:**
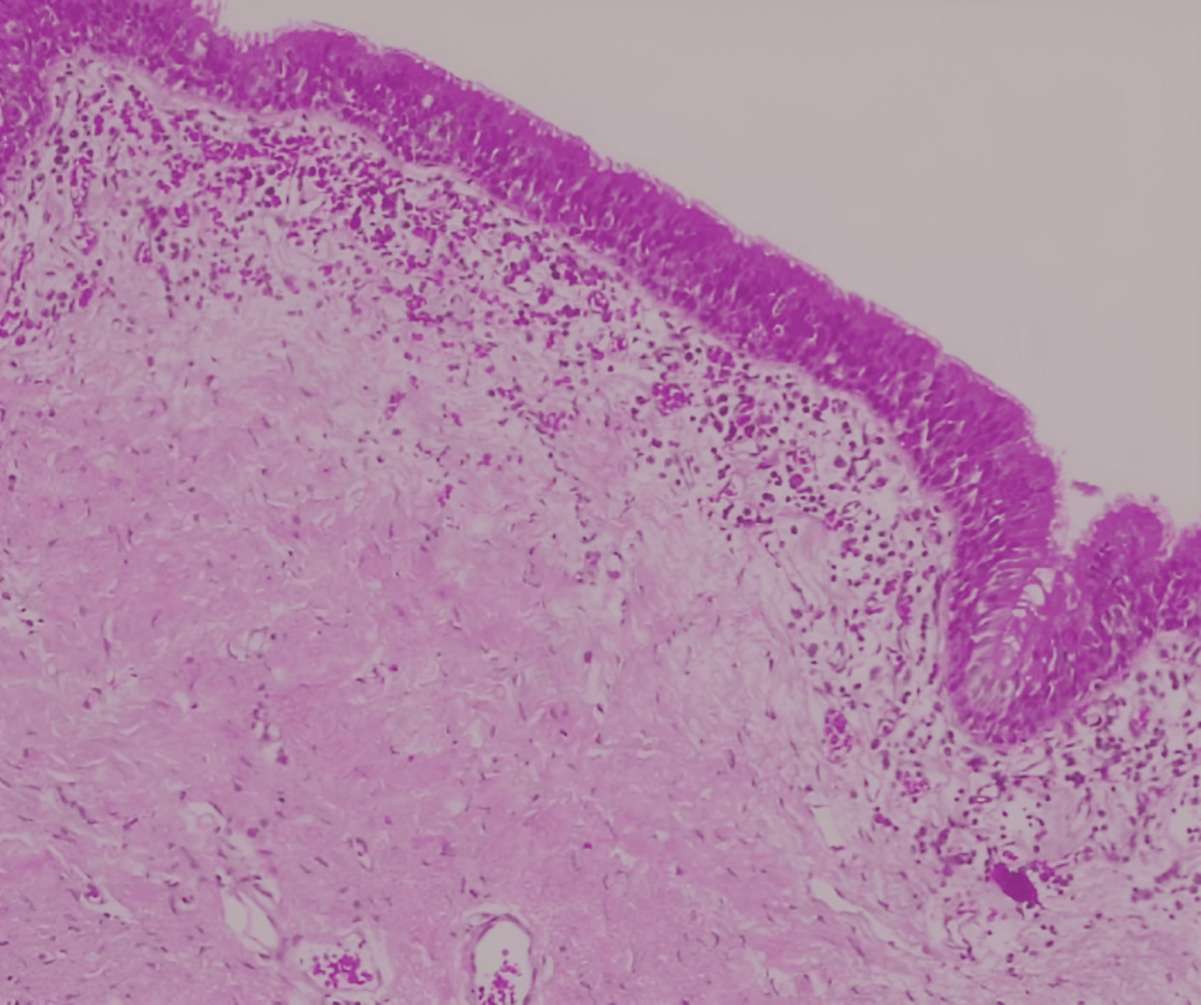
Histopathology report Photomicrograph showing the cystic histology of the lesion wall, lined by respiratory epithelium. Below the epithelium, a large amount of lymphoplasmacytic infiltrate is observed (H&E, 10x magnification). The cystic wall also features bronchial epithelium, lymphoid follicles, a thick muscular wall without cartilage, and areas of squamous metaplasia.

The pathology report confirmed the diagnosis of a bronchogenic cyst (Figure 3). The report described a cystic wall lined with bronchial epithelium, featuring lymphoid follicles, a thick muscular wall without cartilage, and areas of squamous metaplasia. Additionally, the evaluation of the resected pulmonary parenchyma revealed bronchiectasis with hyphae and conidia suggestive of an aspergilloma, as well as pneumonia in various stages of organization.

**Figure 4:**
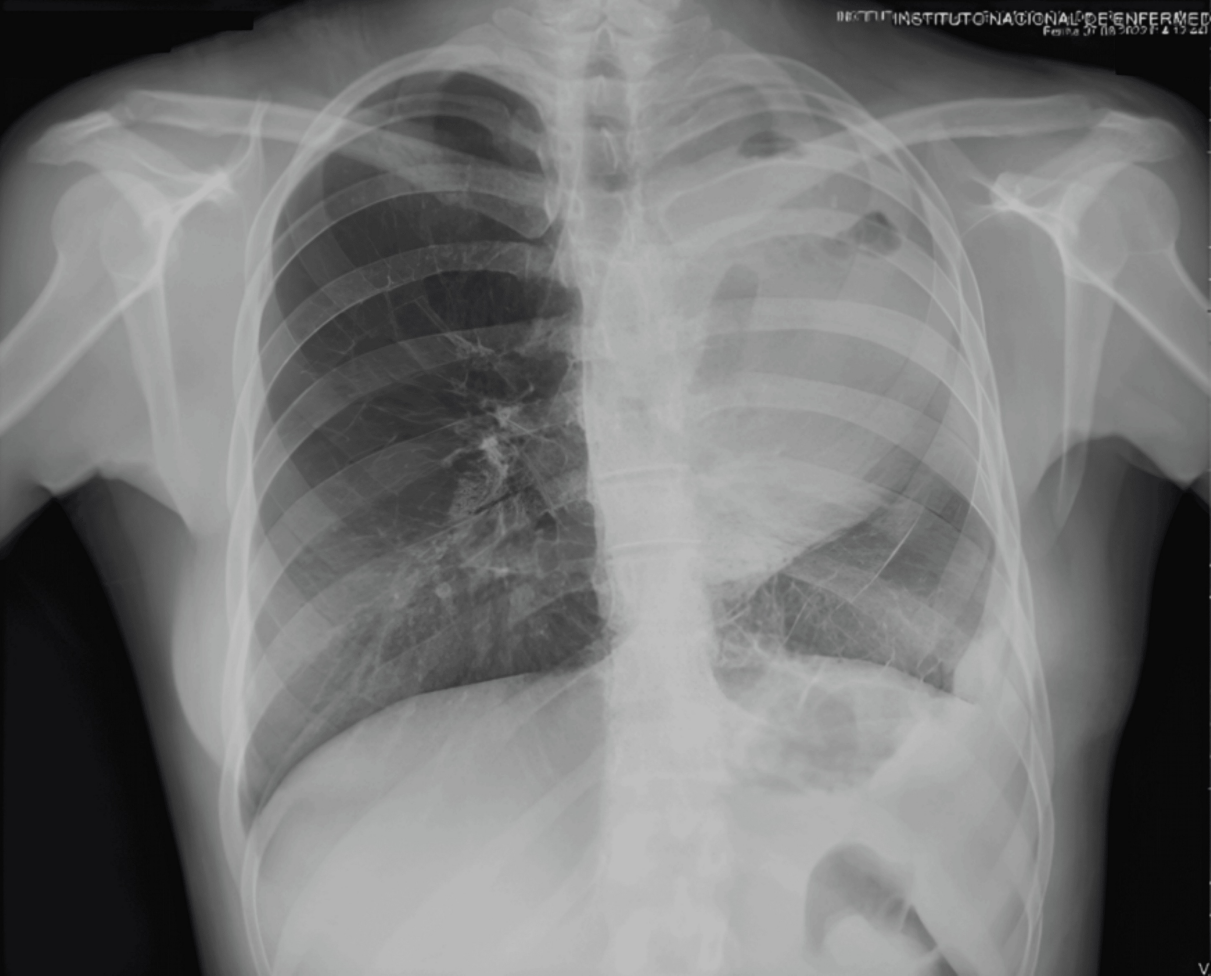
Postoperative chest X-ray

The patient had a satisfactory postoperative course, although dopamine infusion was initially required, and antibiotic therapy with piperacillin/tazobactam was started due to the isolation of *Klebsiella pneumoniae* from a sputum sample. Postoperative chest X-ray showed evidence of cardiac displacement, but serial electrocardiographic and cardiac enzyme monitoring revealed no cardiac abnormalities (Figure 4). Following the discovery of the absent pericardial sac, a cardiology evaluation was performed. An echocardiogram showed no hemodynamic changes, with the left cavities exhibiting adequate contractility. There was no obstruction of the ascending aorta or pulmonary artery, ruling out heart failure.

Bronchoscopies were performed on the third and 10th postoperative days due to left lower lobe atelectasis. These procedures revealed abundant secretions, which were cleared through aspiration, with no leakage observed from the bronchial stump. Dopamine infusion was discontinued on the fifth postoperative day, and pleural drainage was removed one week after surgery. The patient showed satisfactory progress with no signs of low cardiac output and was discharged after two weeks.

## Discussion

Bronchogenic cysts are rare congenital malformations with an incidence of approximately 1 in every 42,000 to 68,000 hospitalizations [1]. They result from abnormal budding of the pulmonary diverticulum or the tracheobronchial tree during embryonic development [3]. Histologically, bronchogenic cysts are characterized by respiratory epithelium with areas of cartilage, smooth muscle, and glands [2]. Symptoms vary based on size and location, but commonly include thoracic pain, dyspnea, and dysphagia, often due to compression of the esophagus or airway. Diagnosis is typically made using CT [3,4]. Traditionally, treatment involves surgical resection of the cyst via a thoracotomy approach.

Recent advancements in minimally invasive thoracic surgery now enable cyst resection through VATS, or robotic-assisted thoracic surgery [1,4]. In this case, resection was performed via VATS lobectomy, yielding favorable outcomes and allowing the patient to be discharged after a one-year follow-up. She continues to receive follow-up care from the cardiology service.

The literature notes various associations between bronchogenic cysts and other congenital malformations. However, the link between bronchogenic cysts and pericardial agenesis is exceedingly rare, with only about 17 reports before this one [4,5]. Pericardial agenesis itself is among the rarest cardiac malformations, with a prevalence of 0.044% reported in a surgical series [6].

This rare pericardial malformation typically involves a complete absence of the left pericardium, which is linked to embryonic atrophy of the common cardinal vein (Cuvier’s canal). This condition results in inadequate blood flow to the pericardial pleural membrane, leading to its agenesis [5]. Symptomatic patients may experience fatigue, thoracic pain, or cardiac conduction disorders [7]. Diagnosis primarily relies on MRI and CT, which can reveal the extent of the defect and any associated features, such as herniation, a prominent main pulmonary artery, interposition of the left lung between the great vessels, adherence of the right pericardium to the anterior thorax, and heart displacement [6].

The relationship between bronchogenic cysts and pericardial agenesis has been explored by authors such as Rusby and Sellors. Their 1945 study suggested that early atrophy of Cuvier’s canal was linked to anomalous development of adjacent pulmonary segments, which could extend cranially and present as a superior lobe bronchogenic cyst [5,8]. Similar findings were observed in a case series by Jones, which aligns with the observations in the current report [5].

Treatment for patients with pericardial agenesis is generally reserved for those with hemodynamic compromise or partial agenesis. Recommended strategies, as outlined by Mukerjee, include atrial appendectomy to prevent herniation, division of adhesions to alleviate painful episodes, and pericardioplasty [9]. Despite these interventions, overall survival rates have not shown significant differences compared to the general population [7]. In the case presented, the pericardial defect was discovered incidentally during surgery. Although the heart was displaced due to the superior lobectomy, this did not lead to hemodynamic deterioration. Consequently, conservative management was chosen. The patient continues to be monitored by the cardiology department.

## Conclusions

This case report describes and analyzes the rare association between a bronchogenic cyst and pericardial agenesis. The linkage between these two conditions is infrequent, and while most patients with such abnormalities may be asymptomatic, some present clinically relevant issues. In cases where pericardial agenesis leads to hemodynamic instability, surgical intervention is warranted.
